# Effectiveness of smoking cessation therapies: a systematic review and meta-analysis

**DOI:** 10.1186/1471-2458-6-300

**Published:** 2006-12-11

**Authors:** Ping Wu, Kumanan Wilson, Popey Dimoulas, Edward J Mills

**Affiliations:** 1 Department of Clinical Epidemiology and Biostatistics, McMaster University, Hamilton, Canada; 2 Department of Medicine, University of Toronto, Toronto, Canada; 3 Faculty of Health Sciences, Clinical Epidemiology & Biostatistics, HSC-2C12, 1200 Main Street West, Hamilton, ON L8N 3Z5, Canada

## Abstract

**Background:**

Smoking remains the leading preventable cause of premature deaths. Several pharmacological interventions now exist to aid smokers in cessation. These include Nicotine Replacement Therapy [NRT], bupropion, and varenicline. We aimed to assess their relative efficacy in smoking cessation by conducting a systematic review and meta-analysis.

**Methods:**

We searched 10 electronic medical databases (inception to Sept. 2006) and bibliographies of published reviews. We selected randomized controlled trials [RCTs] evaluating interventions for smoking cessation at 1 year, through chemical confirmation. Our primary endpoint was smoking cessation at 1 year. Secondary endpoints included short-term smoking cessation (~3 months) and adverse events. We conducted random-effects meta-analysis and meta-regression. We compared treatment effects across interventions using head-to-head trials and when these did not exist, we calculated indirect comparisons.

**Results:**

We identified 70 trials of NRT versus control at 1 year, Odds Ratio [OR] 1.71, 95% Confidence Interval [CI], 1.55–1.88, P =< 0.0001). This was consistent when examining all placebo-controlled trials (49 RCTs, OR 1.78, 95% CI, 1.60–1.99), NRT gum (OR 1.60, 95% CI, 1.37–1.86) or patch (OR 1.63, 95% CI, 1.41–1.89). NRT also reduced smoking at 3 months (OR 1.98, 95% CI, 1.77–2.21). Bupropion trials were superior to controls at 1 year (12 RCTs, OR1.56, 95% CI, 1.10–2.21, P = 0.01) and at 3 months (OR 2.13, 95% CI, 1.72–2.64). Two RCTs evaluated the superiority of bupropion versus NRT at 1 year (OR 1.14, 95% CI, 0.20–6.42).

Varenicline was superior to placebo at 1 year (4 RCTs, OR 2.96, 95% CI, 2.12–4.12, P =< 0.0001) and also at approximately 3 months (OR 3.75, 95% CI, 2.65–5.30). Three RCTs evaluated the effectiveness of varenicline versus bupropion at 1 year (OR 1.58, 95% CI, 1.22–2.05) and at approximately 3 months (OR 1.61, 95% CI, 1.16–2.21). Using indirect comparisons, varenicline was superior to NRT when compared to placebo controls (OR 1.66, 95% CI 1.17–2.36, P = 0.004) or to all controls at 1 year (OR 1.73, 95% CI 1.22–2.45, P = 0.001). This was also the case for 3-month data. Adverse events were not systematically different across studies.

**Conclusion:**

NRT, bupropion and varenicline all provide therapeutic effects in assisting with smoking cessation. Direct and indirect comparisons identify a hierarchy of effectiveness.

## Background

Smoking is the leading cause of preventable death in the world[1]. Given the multitude of health benefits of smoking cessation, considerable effort has been focused on identifying mechanisms to assist smokers in quitting. However, smoking cessation is challenging and behavioural interventions have had only modest success [2].

Drug therapy has been increasingly relied upon to assist in smoking cessation. The most common of these has been nicotine replacement therapy [NRT] [3]. More recently, attention has focused on the use of anti-depressant therapy and specifically the agent bupropion[4]. Five new trials have demonstrated the effectiveness of a new agent with a novel mechanism of action, varenicline, in improving cessation rates [5-9].

How effective are pharmacologic smoking cessation therapies? No systematic review and meta-analysis has examined this issue since the availability of information of the newest agent. We conducted a meta-analysis of Randomized Controlled Trials [RCTs] to identify the effectiveness of the various pharmacological interventions in improving cessation rates.

## Methods

### Eligibility criteria

Our primary outcome of interest was smoking cessation at 1 year. Our secondary outcomes were short-term smoking cessation defined as 3 months after initiating treatment or closest available data to that time point, within one month. Our additional secondary outcome evaluated adverse events. We included any RCT of NRT of any delivery method, bupropion or varenicline. We included only RCTs of at least 1 years duration with chemical confirmation of smoking cessation, as there is reason to doubt patient self-report regarding addictions. Methods of assessing smoking cessation also vary from study to study. The most common method is self-report, however, this can have false cessation rates as high as 30%[10]. False reporting is most likely to occur in a trial setting or in assessing smoking status after a medical event. In both situations the smoker is under considerable pressure to quit. Laboratory tests are, therefore, often used to verify smoking status, especially in clinical trials. Methods of biological verification include serum and saliva thiocyanate (SCN), expired carbon monoxide (CO), plasma, saliva and urinary cotinine and plasma and urinary nicotine. Each of these have various strengths and weaknesses[11]. Studies had to report smoking cessation as either sustained abstinence at the time periods or point-prevalence of abstinence. When both outcomes were available, we considered sustained abstinence to be a superior clinical marker of abstinence. We excluded non-RCTs, post-hoc analyses, maintenance therapy, and studies that reported outcomes as self-report.

### Search strategy

In consultation with a medical librarian (PR), we established a search strategy. We searched independently, in duplicate, the following 10 databases (from inception to September 10, 2006): MEDLINE, EMBASE, Cochrane CENTRAL, AMED, CINAHL, TOXNET, Development and Reproductive Toxicology, Hazardous Substances Databank, Psych-info and Web of Science, databases that included the full text of journals (*OVID*, *ScienceDirect*, and *Ingenta*, including articles in full text from approximately 1700 journals since 1993). In addition, we searched the bibliographies of published systematic reviews[3,12,4,19] and health technology assessments[20]. Searches were not limited by language, sex or age.

### Study selection

Two investigators (EM, PW) working independently, in duplicate, scanned all abstracts and obtained the full text reports of records, that indicated or suggested that the study was a RCT evaluating a smoking cessation therapy on the outcomes of interest. After obtaining full reports of the candidate trials (either in full peer-reviewed publication or press article) the same reviewers independently assessed eligibility from full text papers.

### Data collection

Two reviewers (PW, PD) conducted data extraction independently using a standardized pre-piloted form. Reviewers collected information about the smoking cessation intervention tested, the population studied (age, sex, underlying conditions), treatment dosages and dosing schedules, the treatment effect at 1 year and at 3 months, the specific measurement of abstinence (sustained or point-prevalence), and the chemical confirmation methods. Study evaluation included general methodological reporting quality features including allocation concealment, sequence generation, blinding status, intention-to-treat, and appropriate descriptions of loss to follow-up. Quality of reporting could be considered as analogous to methodological quality if one assumes that failure to report on a component of study design (for example blinding) actually indicated that the component was not employed. We entered the data into an electronic database such that duplicate entries existed for each study; when the two entries did not match, we resolved differences through discussion and consensus.

### Data analysis

In order to assess inter-rater reliability on inclusion of articles, we calculated the *Phi *statistic (ϕ), which provides a measure of inter-observer agreement independent of chance[21]. We calculated the Odds Ratios [OR] and appropriate 95% Confidence Intervals [CIs] of outcomes according to the number of events of abstinence reported in the original studies or sub-studies. In circumstances of zero outcome events in one arm of a trial, we added 1 to each arm, as suggested by Sheehe[22]. We first pooled studies of all NRT interventions versus all controls using the DerSimonian-Laird random effects method,[23] which recognizes and anchors studies as a sample of all potential studies, and incorporates an additional between-study component to the estimate of variability[24]. We calculated the I^2 ^statistic for each analysis as a measure of the proportion of the overall variation that is attributable to between-study heterogeneity[25]. Forest plots are displayed for each primary analysis, showing individual study effect measures with 95% CIs, and the overall DerSimmonian-Laird pooled estimate. We then conducted a meta-regression analysis on the NRT studies with predictors of heterogeneity including the following covariates: placebo control; reporting of sequence generation; reporting of allocation concealment; use of gum or patch; and, method of chemical confirmation of abstinence. When the meta-regression indicated heterogeneity, we conducted alternative sensitivity tests using z-tests to determine differences between the studies reporting the covariates to the pooled all-studies effect size. We additionally conducted separate pooled analyses of NRT versus placebo, gum versus control and patch versus control. We conducted all analyses at 1 year and also at 3 months. For bupropion trials, we pooled all bupropion trials (standard and sustained-release) versus all controls and conducted a meta-regression analysis using the following covariates: placebo control; reporting of sequence generation; reporting of allocation concealment; method of chemical confirmation of abstinence; and plans to quit. We conducted separate meta-regression analyses and calculated the relevant ORs for the covariates as the exponent of the point estimates[26]. The point estimates and 95% CI around denote the expected change in the pooled effect size when the covariates are considered. The OR of the point estimates and 95% CI confer the likelihood of the covariate affecting individual trial outcomes. We additionally pooled all placebo-controlled trials and evaluated effect sizes at 1 year and at 3 months. For head-to-head trials of bupropion versus NRT, we conducted pooled random-effects analyses at 1 year and at 3 months. For varenicline trials, we conducted pooled random-effects analyses of varenicline versus placebo at 1 year and at 3 months and for head-to-head trials of varenicline versus bupropion at 1 year and at 3 months. Head-to-head trials provide the strongest inferences regarding intervention superiority[27]. However, in the absence of head-to-head trials of varenicline versus NRT, we conducted indirect comparisons of these interventions versus placebo using methods described by Bucher et al and conducted z-tests to confirm[28]. This method maintains the randomization from each trial and compares the summary estimates of pooled interventions with CIs. We calculated adverse events, where reported, using Peto's Odds Ratio [OR] with 95% CIs[29]. Analyses were conducted using StatsDirect (version 2.5.2) and Comprehensive Meta-analysis (version 2).

## Results

**Figure 1:**
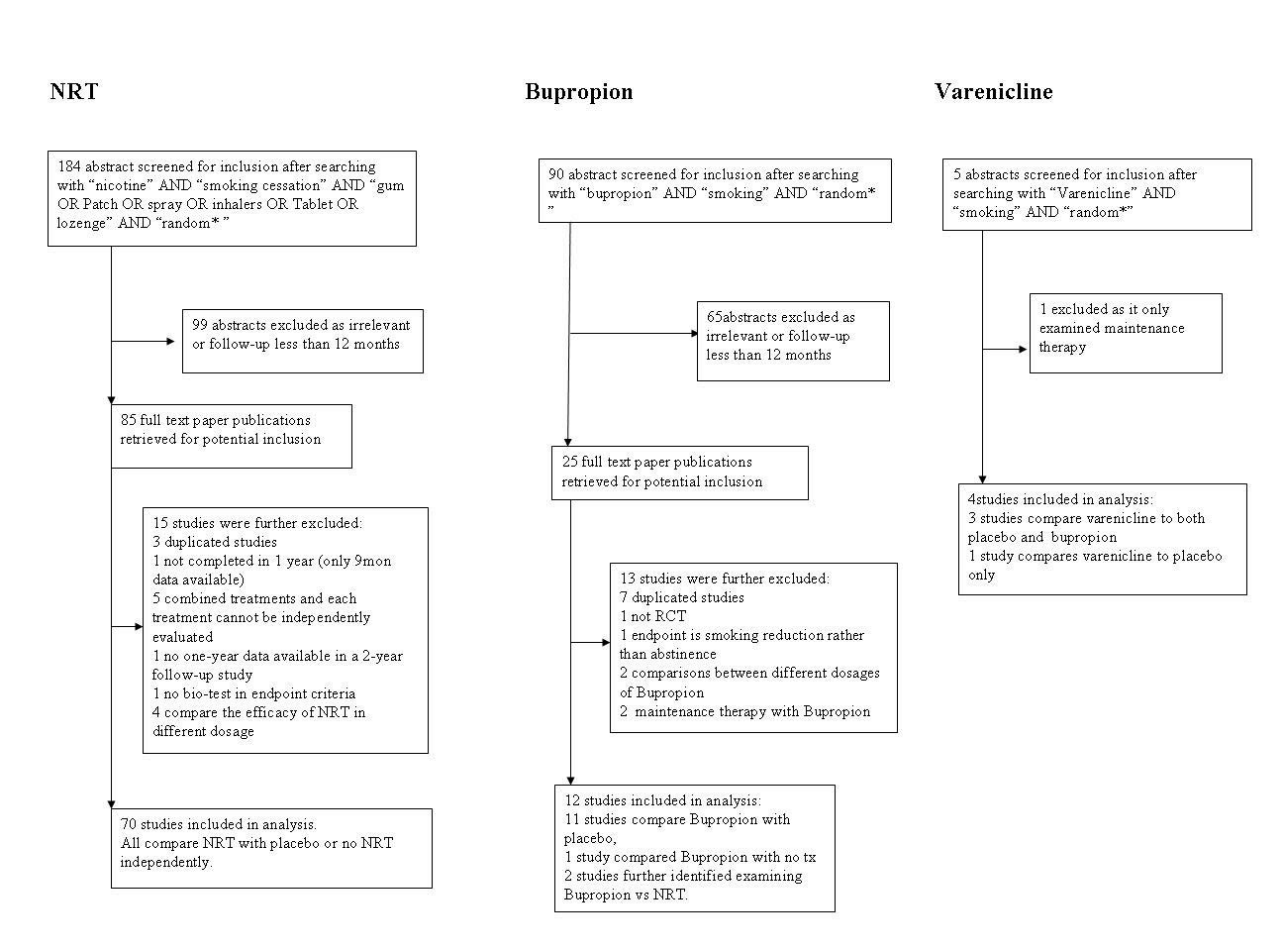
Flowcharts of study searches.

We found 70 RCTs examining NRT versus control interventions (See Figure 1 and Additional File 1), [30-99] 49 of which compared NRT to placebo [30,32,34,35,38,39,43,44,46,48,50,54,56-59,62,64-69,71-82,84-87,89-91,93,94,96,98,99] thirty one studies compared NRT to other controlled groups (See table 1) [31,33,36,37,40-42,45,49,51-53,55,61,63,70,83,88,92,95,97] and 1 study uses both placebo and no intervention as control group[47].

Thirty-three studies evaluated NRT gum [30-49,51-53,55,61,63,64,71,83,87,95,96,98,99], 23 evaluated NRT patch [50,56,57,60,62,65,67-70,72,76,78-80,82,84,86,88,90,92,94,97]. The remaining studies evaluated the efficacy of nicotine inhalers, nasal spray or lozenges [54,58,59,66,73-75,77,81,85,89,91,93].

All of the studies provided sufficient details to evaluate NRT versus control at 1 year [30-99]. Fifty-nine provided sufficient details to evaluate NRT versus control at or about 3 months[30-33,36,37,39-41,44,47,49-54,56,43-61,63-86,88-96,98,99].

We also found 11 studies evaluating bupropion versus placebo [5,6,8,82,100-106] and one RCT evaluating bupropion with no intervention[97] (See Figure 1 and Additional File 2). Further, 2 of these evaluated bupropion versus NRT [82,97].

Finally, we identified 4 studies evaluating varenicline versus placebo (See Figure 1 and Additional File 3) [5,6,8,9]. Of these, 3 also evaluated varenicline versus bupropion[5,6,8]. Agreement on original inclusion of all trials was excellent (ϕ = 0.88).

### Quality of methodological reporting

Of the 70 trials assessing NRT versus controls, studies were varied in reporting important methodological features, including: sequence generation (22/70), allocation concealment (11/70), blinding status (64/70), appropriate blinding (45/70), intention-to-treat (67/70), and appropriate descriptions of loss to follow up (44/70).

Studies assessing bupropion versus controls were similarly varied in reporting, including: sequence generation (4/12), allocation concealment (4/12), blinding status (11/14), intention to treat (12/12) and appropriate descriptions of loss to follow up (10/12).

The 4 studies assessing varenicline appropriately reported the methodological criteria, except 1 study that did not report the method of sequence generation[9].

### Meta-analysis

#### NRT

**Figure 2:**
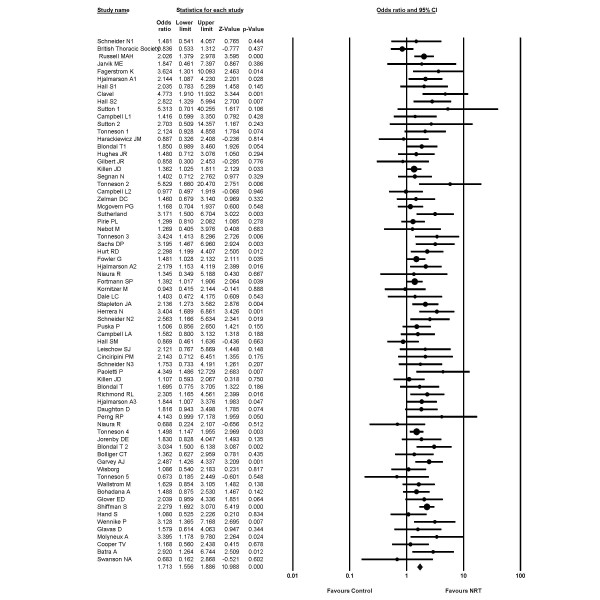
Random Effects Meta-Analysis. NRT vs. Controls at 12 months. I^2 ^= 26.5%.

We combined 70 trials (total n = 28,343) assessing NRT versus controls at 1 year. The pooled OR of smoking cessation favored NRT over controls (OR 1.71,95% CI, 1.55–1.88, P =< 0.0001, I^2 ^= 26.5%, Heterogeneity P = 0.02, See Figure 2). This was consistent when evaluating only placebo controlled NRT trials (49 trials, n = 21,512, OR 1.78, 95% CI, 1.60–1.99, P =< 0.0001, I^2 ^27.4%, Heterogeneity P = 0.04) or when evaluating with cessation as sustained abstinence (52 trials, total n = 22,704, OR 1.72 95% CI, 1.54–1.93, P =< 0.0001, I^2 ^= 29.4%, Heterogeneity P = 0.02) or point prevalence (31 trials, n = 10,686, OR 1.53, 95% CI, 1.30–1.81, P = 0.01, I^2 ^= 46%, Heterogeneity P = 0.01). This was also consistent whether one evaluated NRT gum (33 trials, total n = 12,245, OR 1.60, 95% CI, 1.37–1.86, P =< 0.0001, I^2 ^= 35.8%, Heterogeneity P = 0.02) or NRT patch (23 trials, total n = 11,108, OR 1.63, 95% CI, 1.41–1.89, P =< 0.0001, I^2 ^= 12.3%, Heterogeneity P = 0.24).

**Figure 3:**
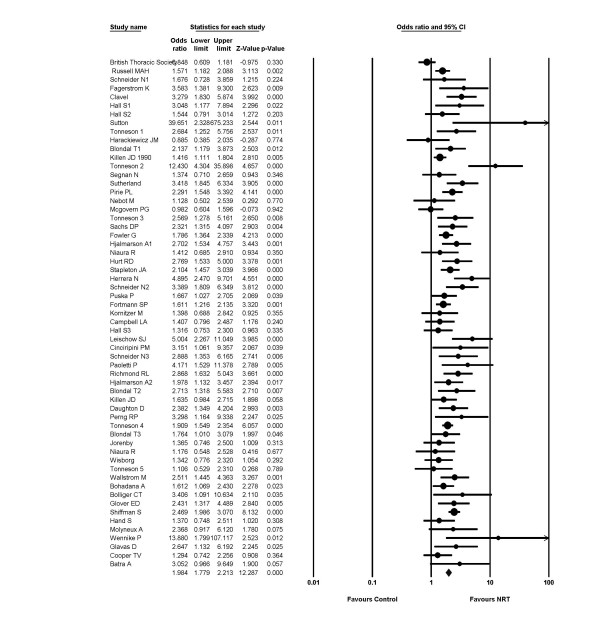
Random Effects Meta-Analysis. NRT vs. Controls at 3 months. I^2 ^= 55.5%.

Fifty-nine trials (total n = 25,294) provided sufficient details to determine short-term effects of NRT on smoking cessation, as determined at 3 months. The pooled OR of the 59 trials was 1.98 (95% CI, 1.77–2.21, P =< 0.0001, I^2 ^= 55.5%, Heterogeneity P =< 0.0001, See Figure 3). The superiority of NRT over controls was consistent whether one evaluated placebo-controlled trials (42 trials, total n = 19,216, OR 2.11, 95% CI, 1.86–2.40, P =< 0.0001, I^2 ^= 57.6%, Heterogeneity P =< 0.001), sustained abstinence (41 trials, total n = 19,854, OR 2.04, 95% CI, 1.80–2.31, P =< 0.0001, I^2 ^= 58%, Heterogeneity P =< 0.0001) or point prevalence at 3 months (21 trials, total n = 6,453, OR 1.78, 95% CI, 1.47–2.14, P =< 0.0001, I^2 ^= 42.4, Heterogeneity P = 0.004). Studies assessing gum versus controls at 3 months (24 trials, total n = 9,347) yielded an OR of 1.71 (95% CI, 1.41–2.07, P =< 0.0001, I^2 ^= 62%, Heterogeneity P =< 0.0001) and studies assessing patch versus controls (21 trials, total n = 10,957) yielded an OR of 1.93 (95% CI, 1.67–2.24, P =< 0.0001, I^2 ^= 35%, Heterogeneity P = 0.05).

#### Bupropion

**Figure 4:**
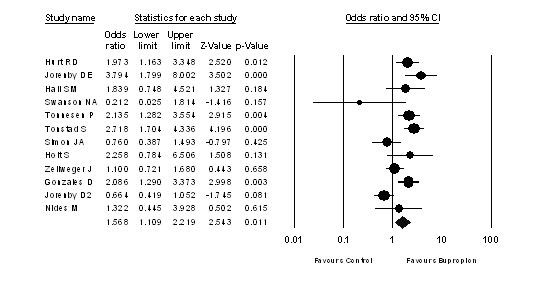
Random Effects Meta-Analysis. Bupropion vs. Controls at 12 months. I^2 ^= 71.5%.

We evaluated the effect of bupropion on smoking cessation relative to adequate controls at 1 year in 12 trials (total n = 5,228, See Figure 4). The pooled OR was 1.56 (95% CI, 1.10–2.21, P = 0.01, I^2 ^= 71.5%, Heterogeneity P =< 0.001). This effect was consistent whether examining placebo-controls (11 trials, total n = 5,148, OR 1.64, 95% CI, 1.16–2.30, P =< 0.001, I^2 ^= 72%, Heterogeneity P = 0.001), sustained abstinence (11 trials, total n = 4,613, OR 1.52, 95% CI, 1.04–2.23, P =< 0.0001, I^2 ^= 73.6%, Heterogeneity P = 0.0001), or point prevalence (10 trials, total n = 4,845, OR 1.56, 95% CI, 1.13–2.16, P =< 0.0001, I^2 ^= 75.1%, Heterogeneity P =< 0.0001).

**Figure 5:**
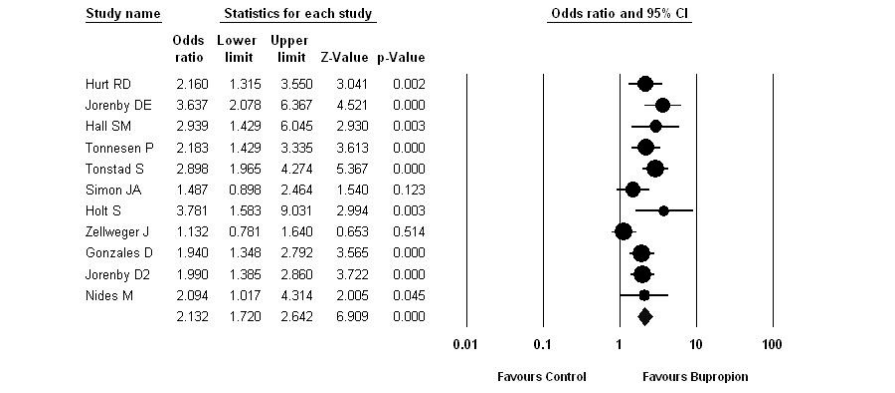
Random Effects Meta-Analysis. Bupropion vs. Controls at 3 months. I^2 ^= 53.6%.

When we evaluated the effect of bupropion on placebo at 3 months (11 trials, total n = 5,148), the OR was 2.13 (95% CI, 1.72–2.64, P =< 0.0001, I^2 ^= 53.6%, Heterogeneity P = 0.01, See Figure 5). This effect was consistent across sustained abstinence measures (8 trials, total n = 4,143, OR 2.18, 95% CI, 1.67–2.86, P =< 0.0001, I^2 ^= 63.5%, Heterogeneity P = 0.008) and point prevalence measures (9 trials, total n = 4,765, OR 2.11, 95% CI, 1.77–2.52, P =< 0.0001, I^2 ^= 38.8%, Heterogeneity P = 0.10).

#### Varenicline

**Figure 6:**
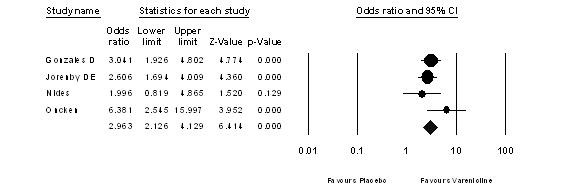
Random Effects Meta-Analysis. Varenicline vs. Placebo at 12 months. I^2 ^= 20.5%.

**Figure 7:**
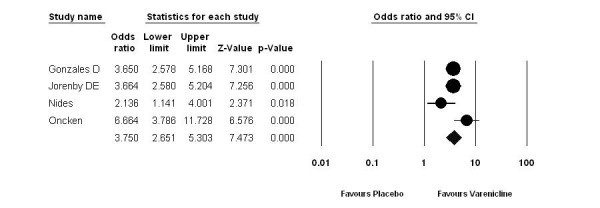
Random Effects Meta-Analysis. Varenicline vs. Placebo at 3 months. I^2 ^= 57.7%.

We pooled 4 studies assessing the effect of varenicline versus placebo at 1 year (total n = 2,528, See Figure 6). The pooled OR is 2.96 (95% CI, 2.12–4.12, P =< 0.0001, I^2 ^= 20.5%, Heterogeneity P = 0.20). This effect was consistent with short-term cessation effects (4 trials, total n = 2,528, OR 3.75, 95% CI, 2.65–5.30, P =< 0.0001, I^2 ^= 57.7%, Heterogeneity P = 0.06, See Figure 7). We explained heterogeneity in this analysis through the inclusion of the dose-ranging studies[8,9].

### Comparisons

**Figure 8:**
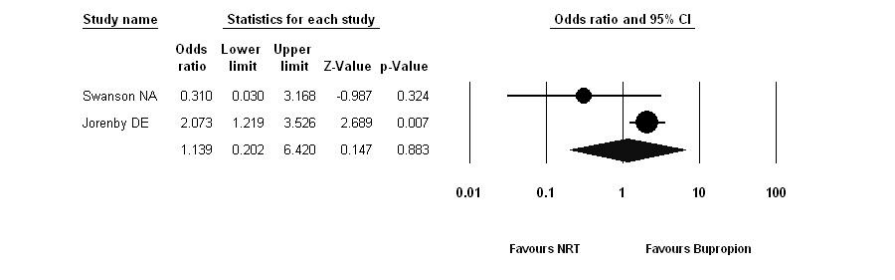
Random Effects Meta-Analysis. NRT vs. Bupropion at 12 months. I^2 ^= 59%.

**Figure 9:**
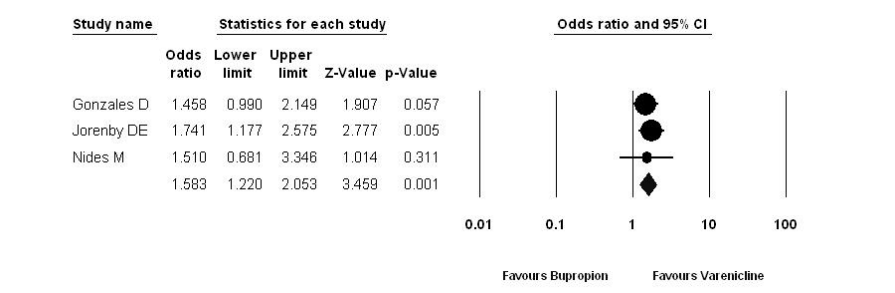
Random Effects Meta-Analysis. Varenicline vs. Bupropion at 12 months. I^2 ^= 0%.

**Figure 10:**
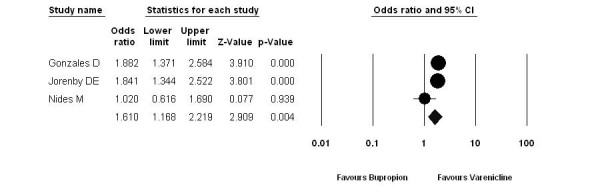
Random Effects Meta-Analysis. Varenicline vs. Bupropion at 3 months. I^2 ^= 56.1%.

Two trials evaluated the superiority of NRT versus bupropion at 1 year [82,97] (total n = 548, See Figure 8) and found a pooled OR of 1.14 (95% CI, 0.20–6.42, P = 0.88, I^2 ^= 59%, Heterogeneity P = 0.11. Only 1 trial provided details on cessation rates at 3 months and favored bupropion (OR 2.66, 95% CI 1.70–4.15, P =< 0.001)[82]. Three trials evaluated the effectiveness of varenicline versus bupropion at 1 year[5,6,8] and yielded a pooled OR of 1.58 (95% CI, 1.22–2.05, P = 0.001, I^2 ^= 0%, Heterogeneity P = 0.81, See Figure 9) in favor of varenicline. These same trials provided consistent data at 3 months (OR 1.61, 95% CI, 1.16–2.21, P = 0.004, I^2 ^= 56.1%, Heterogeneity P = 0.10, See Figure 10).

**Figure 11:**
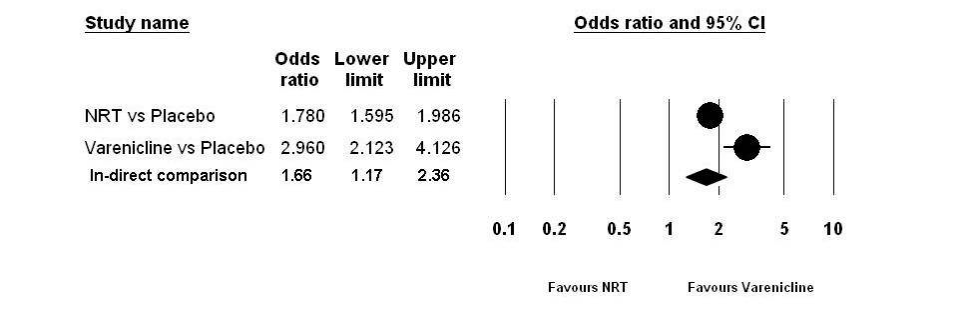
**Indirect comparison between Varenicline and NRT vs Placebo at 12 months**. Bucher et al. indirect comparison methods [28].

**Figure 12:**
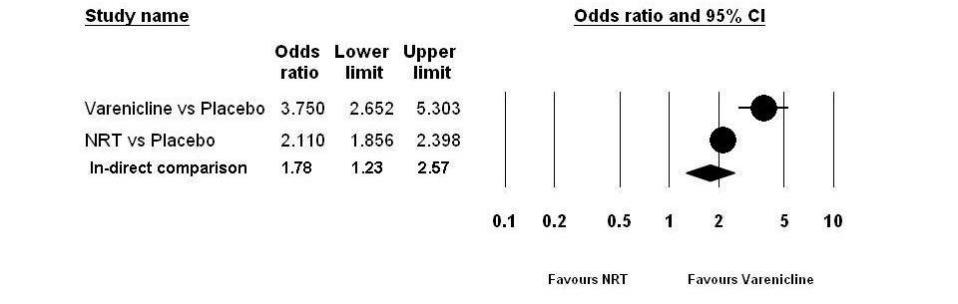
**Indirect comparison between Varenicline and NRT vs Placebo at 3 months**. Bucher et al. indirect comparison methods [28].

Using indirect comparisons[28], we found that bupropion was not superior to NRT when compared to a placebo control at 1 year (OR 0.92, 95% CI 0.64–1.32, test for difference, P = 0.65). This was similar for 3-month data (OR 1.01, 95% CI 0.79–1.29, test for difference 0.94). We found that varenicline was superior to NRT when compared to placebo controls (OR 1.66, 95% CI, 1.17–2.36, test for difference P = 0.004, See Figure 11) or to all controls at 1 year (OR 1.73, 95% CI 1.22–2.45, test for difference P = 0.001). This was also the case when we examined 3-month data for placebo controls (OR 1.78, 95% CI, 1.23–2.57, test for difference P = 0.002, See Figure 12) or all controls (OR 1.89, 95% CI, 1.31–2.73, test for difference P = 0.0006).

### Meta-regression

We anticipated variable between-study heterogeneity, considering the interventions used, methodological issues and measurement tools. Table 1 displays the covariates predicting heterogeneity in the primary outcomes of the NRT analysis using meta-regression. In this analysis, significant predictors of heterogeneity included: allocation concealment, use of NRT gum; and, methods of chemical confirmation (CO, cotinine, and urine markers). Using sensitivity analysis, only studies (n = 3[49,53,71]) using urine as a marker were significantly different from the pooled estimate (P = 0.03), however, all but 1 of these studies also used CO as a chemical marker (P = 0.5)[49].

We additionally examined covariates in the bupropion trials (See Table 2). We found sequence generation was a significant contributor to heterogeneity. In addition, the chemical marker covariates contributed to heterogeneity. We did not conduct a meta-regression on the varenicline studies, given the small number of studies.

### Adverse events

Additional File 1 displays the common adverse events associated with NRT. Inadequate detail was provided for pooling. For NRT trials, we found that the following adverse events were reported significantly more often in active groups than control groups: mouth or throat irritation (n = 12); skin irritation (n = 11); nausea/vomiting (n = 10); coughing (n = 9); hiccoughs (n = 6); dyspepsia (n = 4); watering of eyes (n = 3); headaches (n = 3); heart palpitations (n = 3); sneezing (n = 3); sleep disturbances and dream abnormalities (n = 2); insomnia (n = 2); rhinitis (n = 2); vertigo (n = 1); taste disturbances (n = 1) and muscle aches (n = 1).

For bupropion trials, the following adverse events were reported significantly more in the active groups than control groups: dry mouth (9 trials[5,6,8,82,102-106], n = 4,885, OR 1.90, 95% CI, 1.50–2.42, P =< 0.0001); insomnia (10 trials[5,6,8,82,101-106], n = 4,775, OR 2.02, 95% CI, 1.53–2.68, P =< 0.0001); gastrointestinal upset (7 trials[5,6,8,82,104-106], n = 4,026, OR 1.34, 95% CI, 1.06–1.70, P = 0.01) and constipation (6 trials[5,6,8,104-106], n = 3,622, OR 2.48, 95% CI, 1.62–3.80, P =< 0.0001). Other severe events associated with trial participants in the active arms were: septic shock; grand mal seizure; sleep disorders; and anxiety. These were single cases and did not achieve significance.

For varenicline trials, the following adverse events were reported significantly more often than in the placebo groups: nausea (4 trials [5,6,8,9], n = 2,506, OR 3.17, 2.35–4.29, P =< 0.0001); flatulence (2 trials[5,9], n = 1,323, OR 2.04, 95% CI, 1.16–3.57, P = 0.01); and, constipation (4 trials[5,6,8,9], n = 2,506, OR 2.57, 95% CI, 1.21–5.45, P =< 0.0001). Other, severe events that occurred in the active groups included: atrial fibrillation, pneumonia, possible stroke, chest pain, and elevated blood pressure. These were, however, single cases and did not achieve significance.

## Discussion

In this study we present the results of three main meta-analyses examining the effectiveness of various pharmacological strategies to improve smoking cessation. Our primary meta-analyses specifically examined the effectiveness of NRT versus control, bupropion versus control, and varenicline versus bupropion or placebo. We demonstrated the consistent effectiveness of each intervention in short-term and long-term smoking cessation.

Our findings confirm the effectiveness of two established pharmacological therapies, NRT and bupropion, to improve cessation rates. The primary new information from this review is the evidence of effectiveness of varenicline. This modality of treatment was identified to be more effective than placebo, bupropion and in indirect comparisons with NRT. However, this meta-analysis consisted of the fewest studies and although the studies were well reported and apparently well conducted, further studies comparing varenicline versus either NRT or bupropion will strengthen inferences about the superiority of this intervention. Nevertheless the initial findings are suggestive of the potential superiority of this latest pharmacological intervention.

This review has several strengths and some limitations that deserve mention. The strengths of this review include the comprehensive search strategy that improved the likelihood of identifying all relevant studies. Duplicate extraction of data reduced the potential for bias in this component of the synthesis process. By limiting this review to randomized trials we ensured that the included studies would have reduced likelihood of systematic error and therefore have high internal validity. Our use of meta-regression to identify sources of heterogeneity in the meta-analyses is a strength and demonstrated that several of the *a priori *chosen covariates were predictors of heterogeneity. While our primary endpoint was one-year cessation, as defined by the study, we evaluated the robustness of these findings through multiple sensitivity analyses which included one-year sustained cessation, a strong predictor of sustained smoking cessation[108,109]. Unlike some meta-analyses and systematic reviews of smoking cessation therapies [3,4,12-19], we included only studies that chemically confirmed the cessation of smoking at the specific time-points. This is a strength of our analysis as we demonstrated that even the different chemical markers for cessation can contribute greatly to between-study differences. Our meta-regression analysis indicates that the type of chemical markers employed yields greater variability of effects between studies over more commonly investigated covariates, such as methodological issues[110].

Limitations of this meta-analysis include the potential for publication bias, specifically the possibility that small negative studies would not be published. We included only published trials so it is possible that other trials have been conducted and never published. However, it is unlikely that the presence of these studies would have altered the findings of the NRT meta-analysis given the large number of studies included. Similarly, since varenicline is a new agent and the studies synthesized were the first clinical trials evaluating its effectiveness, it is improbable other negative studies exist. We also limited our search to English language databases (although we would include non-English articles if identified) so the possibility of quality studies in other languages does exist. We used both direct and indirect comparisons to evaluate the relative effectiveness of agents. Head-to-head trials provide the strongest inferences regarding intervention superiority[27]. However, in the absence of head-to-head trials, specifically NRT versus varenicline, we conducted indirect comparisons. We used the indirect comparison method proposed by Bucher et al., which respects the principle of randomization between trials [28]. The utility of indirect comparisons is debatable and some evidence suggests that indirect comparisons may provide misleading measures of superiority, although the direction of bias cannot be assumed until direct comparison trials have been completed[111]. Figure 8 displays an interesting methodologic outcome related to choosing random-effects models over a fixed-effects model for meta-analyses. The pooled OR of the two head-to-head trials is a non-significant difference of 1.14 (95% CI, 0.20–6.42, P = 0.88) when using the random effects model, as planned in our protocol [82,97]. However, had we used a fixed effects model, this would have resulted in a significant pooled OR of 1.84 (95% CI, 1.11–3.06, P = 0.02). We believe that this is due to the very small number of trials included (n = 2) [82,97], where one trial is much larger than the other (n of 488 compared to an n of 60). Finally, our meta-analyses did not examine in detail other aspects of therapy, such as compliance and tolerance, which may influence real life clinical effectiveness.

There are several important implications of these meta-analyses. Identifying effective mechanisms to improve smoking cessation is essential. Smoking is the number one preventable cause of death in the world[1]. Approximately one out of every two long-term smokers will die of a smoking related death[112]. Furthermore, smoking cessation has been clearly demonstrated to reduce the likelihood of future morbidity. Smoking cessation in individuals with chronic obstructive lung disease reduces the deterioration of FEV_1_[113]. Smoking cessation also reduces the likelihood of developing several smoking related cancers[109]. Smoking cessation in patients with coronary artery disease has been consistently shown to be associated with dramatically improved survival. Observational studies demonstrate that smokers who quit smoking after a myocardial infarction have mortality rates approximately 40% less than those that continue to smoke, suggesting that smoking cessation could be one of the most effective therapies to improve survival in this group of patients[114]. Former smokers have also been demonstrated to have considerably lower rates of cerebrovascular disease than ongoing smokers[115].

## Conclusion

The findings of this review point the direction for future studies. It is interesting that the three modalities examined have distinct biological mechanisms of action. NRT presumably works by reducing symptoms of nicotine withdrawal, thereby increasing the likelihood of smoking cessation. Bupropion is a weak dopamine and nor-epinephrine reuptake inhibitor. One of the primary symptoms of smoking cessation has been depressive symptoms and it has been hypothesized that smokers may be increasing central dopamine levels by reducing monoamine oxidase inhibitor activity[116]. The mechanism of action of bupropion, therefore, may be to maintain central levels of dopamine through the process of cessation, although its effectiveness has been identified to be independent of symptoms of depression[102]. Varenicline is a nicotinic acetylcholine receptor partial agonist. The authors of one the studies demonstrating its efficacy comment that "Partial agonists at this (receptor) could stimulate the release of sufficient dopamine to reduce craving and withdrawal while simultaneously acting as a partial antagonist by blocking the binding and consequent reinforcing effects of smoked nicotine[5]." Regardless of the exact mechanism of action of the three modalities, it is clear they are distinct and suggest the possibility of combination therapy or therapy targeted on the particular type of symptoms experienced during cessation. Future studies could examine these options, given that despite the effectiveness of these therapies rates of smoking remain high at one year in the treatment groups [82]. Furthermore, given the benefits described of smoking cessation as secondary prevention, the use of these cessation modalities in patients with active smoking related disease warrants further study [117]. Future studies should further examine the safety and effectiveness in reducing morbidity and mortality of all three of these modalities in patients with active smoking related disease.

## Competing interests

This study was supported by an unrestricted educational grant from Pfizer Global Solutions and the Canadian Institutes of Health Research (CIHR). The terms of the support from Pfizer and CIHR included freedom for authors to reach their own conclusions, and an absolute right to publish the results of their research, irrespective of any conclusions reached.

## Authors' contributions

PW conducted the systematic searches, abstracted data, analyzed the data and contributed to the manuscript.

PD abstracted data, analyzed the data and contributed to the manuscript.

KW conceived the study, abstracted data, analyzed the data and contributed to the manuscript.

EM conceived the study, conducted the searches, abstracted data, analyzed the data and contributed to the manuscript.

All authors have read and approved the final manuscript.

## Pre-publication history

The pre-publication history for this paper can be accessed here:



## Supplementary Material

Additional File 1 Characteristics of NRT RCTs. Word file displays specific study details Click here for file

Additional File 2 Characteristics of Bupropion RCTs. Word file displays specific study details Click here for file

Additional File 3 Characteristics of Varenicline RCTs. Word file displays specific study details Click here for file

## Figures and Tables

**Table 1:** Univariable meta-regression of NRT studies

**Covariates**	**Point estimate**	**Lower 95% limit**	**Upper 95% limit**	**OR**	**Lower 95% limit**	**Upper 95% limit**	**p-value**
Placebo	0.13	-0.03	0.31	1.13	0.97	1.36	0.12
Sequence generation	-0.15	-0.32	0.006	0.86	0.72	1.00	0.06
Allocation concealment	-0.20	-0.39	0.01	0.81	0.67	1.01	0.03
Gum	-0.18	-0.32	-0.02	0.83	0.72	0.98	0.02
Patch	0.0007	-0.15	0.16	1.00	0.86	1.17	0.99
CO	0.52	0.24	0.80	1.68	1.27	2.22	0.0002
Cotinine	-0.21	-0.37	-0.05	0.81	0.69	0.95	0.007
Serum	-0.36	-0.75	0.01	0.69	0.47	1.01	0.06
Urine	-0.36	-0.70	-0.02	0.69	0.49	0.98	0.03
Planned to quit	0.12	-0.02	0.27	1.12	0.98	1.30	0.10

**Legend**. The point estimate and 95% CIs estimate the unit change in the effect size, whenever the predicted covariate is present. The OR for the point estimates and 95% CI denote the likelihood of covariate affecting the trial effect size.

**Table 2:** Univariable meta-regression of bupropion studies.

**Covariates**	**Point estimate**	**Lower 95% limit**	**Upper 95% limit**	**OR**	**Lower 95% limit**	**Upper 95% limit**	**p-value**
Placebo	2.00	-0.14	4.15	7.38	0.86	63.4	0.07
Sequence generation	-0.46	-0.84	-0.08	0.63	0.43	0.92	0.01
Allocation concealment	-0.20	-0.55	0.14	0.81	0.57	1.15	0.25
CO	0.76	0.06	1.46	2.13	1.06	4.30	0.03
Cotinine	-0.76	-1.46	-0.06	0.46	0.23	0.94	0.03
Planned to quit	0.13	-0.22	0.48	1.13	0.80	1.61	0.46

**Legend**. The point estimate and 95% CIs estimate the unit change in the effect size, whenever the predicted covariate is present. The OR for the point estimates and 95% CI denote the likelihood of covariate affecting the trial effect size.
