# Supplementary material for: Effectiveness of smoking cessation therapies: a systematic review and meta-analysis
Source: BMC Public Health. 2006 Dec 11;6:300. doi: 10.1186/1471-2458-6-300 (PMC1764891; doi:10.1186/1471-2458-6-300)
Supplement: Additional File 1 — Characteristics of NRT RCTs. Word file displays specific study details [file 1471-2458-6-300-S1.doc]

| **Year** Additional File 1. | **Author** | **Country** | **Characteristic of the patient (cig/day)**  **(*: Mean or median)** | **Smoking history (year)**  **(*: Mean or median)** | **Intervention** | **Dosage**  **(mg / per intervention)** | **TX. in each studies and the number in each group** | | | | **Duration of the treatment** | **Number in analysis** | | **Side effect** |
| --- | --- | --- | --- | --- | --- | --- | --- | --- | --- | --- | --- | --- | --- | --- |
|  |  |  |  |  |  |  | **Treatment in intervention group** | **N** | **Treatment in control group** | **N** |  | **Number in intervention** | **Number in control** |  |
| 1983 | British Thoracic Society | England | 24* | >10  (88%people) | gum | 2 | Gum | 410 | Placebo | 412 | 3-6mon | 410 | 412 | Bad taste, nausea, sore throat |
|  |  |  |  |  |  |  | Verbal advice | 395 | Verbal advice + booklet | 401 | Not included | | | |
| 1983 | Russell MA | England | 17.5* | na | gum | 2 | Gum | 679 | No gum | 675 | na | 679 | 675 | na |
|  |  |  |  |  |  |  | / |  | Not any intervention | 584 | Not included | | | |
| 1983 | Schneider NG | USA | >=20 | na | gum | 2 | Gum  +clinic support | 30 | Placebo +clinic support | 30 | na | 43 | 53 | Hiccups, nausea |
|  |  |  |  |  |  |  | Gum +dispensary study | 13 | Placebo +dispensary study | 23 |  |  |  |  |
| 1984 | Jarvik ME | USA | >=20 | na | gum | 2 | Gum | 25 | Placebo | 23 | na | 25 | 23 | na |
| 1984 | Fagerstrom K | Sweden | 19* | na | gum | 2 to 4 | Gum  +Long follow up | 50 | No gum  +Long follow-up | 22 | 3mon | 96 | 49 | na |
|  |  |  |  |  |  |  | Gum  +Short follow up | 46 | No gum +short follow up | 27 |  |  |  |  |
| 1984 | Hjalmarson AI | Sweden | 23* | na | gum | 2 | Gum | 106 | Placebo | 100 | na | 106 | 100 | No sig. dif. |
| 1985 | Clavel | France | >=5 | na | gum | 2 | Gum | 205 | No gum | 222 | 105pieces | 205 | 222 | na |
|  |  |  |  |  |  |  | / |  | Acupuncture | 224 | Not included | | | |
| 1985 | Hall SM | USA | 30.5* | na | gum | 2 | Gum  +Intensive behavioral therapy | 41 | No gum  + Intensive behavioral therapy | 36 | 6mon | 41 | 36 | na |
|  |  |  |  |  |  |  | Gum +low-contact treatment | 42 |  |  | Not included | | | |
| 1987 | Sutton | England | 16* | na | gum | 2 | Gum | 270 | No gum | 64 | 420 pieces | 270 | 64 | na |
| 1987 | Campbell IA | England | na | na | gum | 2 | Gum | 424 | Placebo | 412 | 630 pieces | 424 | 412 | na |
| 1987 | Hall SM | USA | 30* | na | gum | 2 | Gum  + Low contact condition | 36 | Placebo  +Low contact condition | 34 | 12mon | 71 | 68 | na |
|  |  |  |  |  |  |  | Gum  + Behavioral Tx. | 35 | Placebo  + Behavioral Tx. | 34 |  |  |  |  |
| 1988 | Sutton | England | 19* | na | gum | 2 | Gum | 79 | No gum | 82 | 105 pieces | 79 | 82 | na |
| 1988 | Tonnesen | Sweden | >=10 | na | gum | 2 | Gum  (Low-medium dependence G) | 60 | Placebo | 53 | 2-24mon | 60 | 53 | Hiccups |
|  |  |  |  |  |  | 4 | Gum  (High dependence G) | 27 | Gum (2mg) | 33 | Not included | | | |
| 1988 | Harackiewicz JM | USA | >=10 | 17* | gum | 2 | Gum  +Self-help manual | 99 | No gum  +Self-help manual | 52 | 6mon | 99 | 52 | Hiccups, jaw muscle ache, nausea, stomach/GI symptoms, oral soreness |
|  |  |  |  |  |  |  | / |  | No any intervention | 46 | Not included | | | |
| 1989 | Hughes JR | USA | 30* | 19* | gum | 2 | Gum | 210 | placebo | 105 | 3-12mon | 210 | 105 | na |
| 1989 | Gilbert RJ | Canada | >=1 | na | gum | 2 | Gum | 112 | No gum | 111 | 2-3mon | 112 | 111 | na |
| 1989 | Blondal T | Iceland | 21g * | na | gum | 4 | Gum | 92 | Placebo | 90 | 3mon | 92 | 90 | Hiccups |
| 1990 | Killen JD | USA | na | na | gum | 2 | Gum (ad lib) | 301 | Placebo | 309 | 8wks | 600 | 309 | na |
|  |  |  |  |  |  |  | Gum (fixed schedule) | 299 | No gum | 309 |  |  | 309 |  |
| 1991 | Segnan N | Italy | na | na | gum | na | Gum | 294 | No gum | 275 | 3mon | 294 | 275 | na |
|  |  |  |  |  |  |  | Counseling | 62 | spirometry | 292 | not included | | | |
| 1991 | Tonnesen | Sweden | >=10 | >=3 | patch | 15 | Patch | 145 | Placebo | 144 | 12wks | 145 | 144 | skin irritation, nausea, vertigo |
| 1991 | Campbell IA | England | na | na | gum | 2 to4 | Gum | 107 | Placebo | 105 | 3mon | 107 | 105 | na |
| 1992 | Sutherland G | England | 25* | 22* | Nasal spray | 1 | Spray | 116 | Placebo | 111 | 3-12mon | 116 | 111 | Coughing, eye watering, throat irritation, sneezing |
| 1992 | Zelman DC | USA | >=20 | past 1 year | gum | 2 | Gum  +Skill training | 30 | Rapid smoking  +Skill training | 30 | 3-6mon | 58 | 58 | na |
|  |  |  |  |  |  |  | Gum  +Supporting counseling | 28 | Rapid smoking +support counseling | 28 |  |  |  |  |
| 1992 | McGovern PG | USA | >=25  (58% people) | na | gum | 2 | Gum | 146 | No gum | 127 | 1-12mon | 146 | 127 | na |
| 1992 | Pirie PL | USA | 25* | na | gum | 2 | Gum  +smoking clinic program | 108 | No gum +smoking clinic program | 103 | 2-5mon | 206 | 211 | na |
|  |  |  |  |  |  |  | Gum  +smoking clinic and weight control program | 98 | No gum +smoking clinic and weight control program | 108 |  |  |  |  |
| 1992 | Nebot M | Spain | >=15  (64.2% people) | na | gum | 2 | Gum  +physician counseling | 106 | No gum +physician counseling | 213 | 2-4wks | 93 | 175 | na |
|  |  |  |  |  |  |  | / |  | No gum +nurse counseling | 106 | Not included | | | |
| 1993 | Tonnesen | Sweden | >=10 | >=3 | inhaler | 0.1 | Inhaler | 145 | Placebo | 141 | 3-6mon | 145 | 141 | Mouth and throat irritation, coughing, nausea, headache |
| 1993 | Sachs DP | USA | >=10 | >=3 | patch | 15 | Patch | 113 | Placebo | 107 | 12-18wks | 113 | 107 | No sig. dif. |
| 1994 | Flowler G | England | na | na | patch | na | Patch | 842 | Placebo | 844 | 12wks | 842 | 844 | na |
| 1994 | Hjalmarson A | Sweden | 21* | 26* | Nasal spray | 1 | Spray | 125 | Placebo | 123 | 3-12mon | 125 | 123 | Nasal and throat irritation, runny nose, sneezing, coughing, eyes watering, pounding heart |
| 1994 | Niaura R | USA | 29* | 24* | gum | 2 | Gum | 84 | No gum | 89 | 1-4mon | 84 | 89 | na |
|  |  |  |  |  |  |  |  |  |  |  |  |  |  |  |
| 1994 | Hurt RD | USA | >=20 | Past 1 year | patch | 22 | Patch | 120 | Placebo | 120 | 8wks | 120 | 120 | skin irritation |
| 1995 | Stapleton JA | England | >=15 | na | patch | 15 | Patch | 400 | Placebo | 400 | 18wks | 800 | 400 | skin irritation |
|  |  |  |  |  |  | 25 | Patch | 400 |  |  |  |  |  |  |
| 1995 | Fortmann SP | USA | >=25 | na | gum | 2 | Gum | 262 | No gum | 261 | na | 522 | 522 | na |
|  |  |  |  |  |  |  | Gum  +Material | 260 | No gum +material | 261 |  |  |  |  |
| 1995 | Herrera N | Sweden | >=10 | na | gum | 2 | Gum (in low-medium dependence G) | 76 | Placebo | 78 | 3mon | 76 | 78 | No sig. dif. |
|  |  |  |  |  |  | 4 | Gum (in high dependence G) | 87 | Gum (2mg) | 81 | Not included | | | |
| 1995 | Schneider NG | USA | >=15 | >=2 | Nasal spray | 1 | Spray | 128 | placebo | 127 | 6wks-6mon | 128 | 127 | Throat irritation, coughing, sneezing, runny eyes and nose, palpitation, nausea, feeling good or "high" |
| 1995 | Puska P | Finland | >=10 | >=3 | gum + patch | 15(patch) + 2(gum) | Patch  +gum | 150 | Placebo patch +gum | 150 | 12-18wks | 150 | 150 | No sig.dif. |
|  |  |  |  |  |  |  |  |  |  |  |  |  |  |  |
|  |  |  |  |  |  |  |  |  |  |  |  |  |  |  |
| 1995 | Kornitzer M | Sweden | >=10 | na | gum + patch | 2(gum), 5to15(patch) | Gum  +patch | 149 | Placebo gum +patch | 150 | Not included | | | |
|  |  |  |  |  |  |  | Patch  + placebo gum | 150 | Placebo patch +placebo gum | 75 | 12-24wks | 150 | 75 | No sig.dif. |
| 1995 | Dale LC | USA | >=10 | >=1 | patch | 11 | Patch | 18 | Placebo | 18 | 8wks | 53 | 18 | Skin irritation |
|  |  |  |  |  |  | 22 | Patch | 17 |  |  |  |  |  |  |
|  |  |  |  |  |  | 44-11 | Patch | 18 |  |  |  |  |  |  |
| 1996 | Campbell IA | England | >=1 | Past 1 wk | patch | 7 to 21 | Patch | 115 | Placebo | 119 | 12 week | 115 | 119 | Nausea, skin irritation |
| 1996 | Hall SM | USA | >=10 | 21* | gum | 2 | Gum | 98 | placebo | 103 | 3-6mon | 98 | 103 | na |
| 1996 | Leischow SJ | USA | >=10 | >=3 | inhaler | na | Inhaler | 111 | Placebo | 111 | 3-6mon | 111 | 111 | Coughing, mouth and throat irritation |
| 1996 | Cinciripini PM | USA | >=15 | >=3 | patch | 7 to 14 | Patch | 32 | No patch | 32 | 9-12wks | 32 | 32 | na |
| 1996 | Schneider NG | USA | >=10 | >=3 | inhaler | 13ug/per puff | Inhaler | 112 | Placebo | 111 | 3-6mon | 112 | 111 | Coughing, mouth and throat irritation |
| 1996 | Paoletti P | Italy | >=10 | >=3 | patch | 15 | Patch (in plasma cotinine<=250 G) | 60 | Placebo | 60 | 12-18wks | 60 | 60 | Skin irritation, sleep disturbance |
|  |  |  |  |  |  | 25 | Patch (plasma cotinine>250 G) | 87 | Patch (15mg) | 90 | Not included | | | |
| 1997 | Killen JD | USA | >=10 | na | patch | 7 to 21 | Patch | 103 | Placebo | 104 | 16wks | 212 | 212 | na |
|  |  |  |  |  |  |  | Patch  +video | 109 | Placebo +video | 108 |  |  |  |  |
| 1997 | Blondal T | Iceland | >=1 | na | Nasal spray | 1 | Spray | 79 | Placebo | 78 | 3-12mon | 79 | 78 | Sweating |
| 1997 | Richmond RL | Australia | na | na | patch | 7 to 21 | Patch | 153 | Placebo | 152 | 10wks | 153 | 152 | na |
| 1997 | Hjalmarson A | USA | >=10 | >=3 | inhaler | 13 ng /puff | Inhaler | 123 | Placebo | 124 | 3-6mon | 123 | 124 | Coughing, mouth and throat irritation |
| 1998 | Daughton D | USA | >=20 | 19.3* | patch | 7to21 | Patch | 184 | Placebo | 185 | 10wks | 184 | 185 | na |
| 1998 | Perng RP | Taiwan | >=20 | >=1 | patch | 30 | Patch | 30 | Placebo | 32 | 6wks | 30 | 32 | skin irritation |
| 1999 | Tonnesen | Europe | >=14 | >=3 | patch | 15 | Patch | 716 | Placebo | 714 | 8 wks | 2861 | 714 | Headache, insomnia, palpitation and tachycardia, skin irritation |
|  |  |  |  |  |  |  |  | 715 |  |  | 22wks |  |  |  |
|  |  |  |  |  |  | 25 | Patch | 715 |  |  | 8 wks |  |  |  |
|  |  |  |  |  |  |  |  | 715 |  |  | 22wks |  |  |  |
| 1999 | Jorenby DE | USA | 15 | na | patch | 7 to 21 | Patch | 244 | Placebo | 160 | 8wks | 244 | 160 | Skin irritation, dream abnormalities, insomnia |
|  |  |  |  |  |  |  | Patch +bupropion | 245 | Placebo +bupropion | 244 | Not included | | | |
| 1999 | Blondal T | Iceland | >=1 | >=3 | patch + nasal spray | 1(spray)+patch | Spray  +Patch | 118 | Placebo spray +patch | 119 | 1y | 118 | 119 | Mouth and throat irritation |
|  |  |  |  |  |  |  |  |  |  |  |  |  |  |  |
|  |  |  |  |  |  |  |  |  |  |  |  |  |  |  |
| 1999 | Niaura R | USA | 27.8* | 26.9* | gum | 2 | Gum  +Behavior program | 35 | No gum +behavior program | 32 | 2mon | 66 | 63 | na |
|  |  |  |  |  |  |  | Gum +  Behavior +cue exposure | 31 | No gum + behavior +cue exposure | 31 |  |  |  |  |
| 2000 | Wisborg | Denmark | >=10 | na | patch | 10 to 15 | Patch | 124 | Placebo | 126 | 11wks | 124 | 126 | skin irritation |
| 2000 | Tonnesen | Denmark | >=10 | na | patch + inhaler | 15(patch)+  5(inhaler) | Patch  + inhaler | 115 | Patch (5mg) | 109 | Not included | | | |
|  |  |  |  |  |  |  |  |  | Patch (15mg) | 104 |  |  |  |  |
|  |  |  |  |  |  |  |  |  | Inhaler (5mg) | 118 | 3-9mon | 115 | 118 | na |
| 2000 | Wallstrom M | Sweden | >=10 | >=3 | tablet | 2 | Tablet | 123 | Placebo | 124 | 3-6mon | 123 | 124 | Nausea/ vomiting, dyspepsia, hiccup, gastritis |
| 2000 | Bohadana A | France | >=10 | >=3 | patch + inhaler | 15(patch)+inhaler | Patch  +inhaler | 200 | Placebo patch +inhaler | 200 | 6wks | 200 | 200 | skin irritation |
|  |  |  |  |  |  |  |  |  |  |  |  |  |  |  |
|  |  |  |  |  |  |  |  |  |  |  |  |  |  |  |
| 2000 | Bolliger CT | Switzerland | >=15 | >=3 | inhaler | 13ug/per puff | Inhaler | 200 | Placebo | 200 | 4-18mon | 200 | 200 | Throat irritation, coughing |
| 2000 | Garvey AJ | USA | 5 | na | gum | 2 | Gum | 202 | placebo | 203 | 2mon-1y | 405 | 203 | No sig.dif. |
|  |  |  |  |  |  | 4 | Gum | 203 |  |  |  |  |  |  |
| 2002 | Glover ED | USA | >=10 | >= 3 | tablet | 2 | Tablet | 120 | placebo | 121 | 3-6mon | 120 | 121 | Hiccups, nausea, dyspepsia |
| 2002 | Shiffman S | USA , England | 17* | na | losenge | 2 | Lozenge (in TTFC<30min G) | 459 | Placebo (in TTFC<30min G) | 458 | 6mon | 909 | 909 | Headache, heart burn, hiccup, nausea, coughing |
|  |  |  |  |  |  | 4 | Lozenge (in TTFC>30min G) | 450 | Placebo (in TTFC>30min G) | 451 |  |  |  |  |
| 2002 | Hand S | England | >=1 | na | patch | 10 to 30 | Patch | 136 | No patch | 109 | 3wks | 136 | 109 | na |
| 2003 | Molyneux A | England | >=1 | 33* | 1 in 5(all) | 2,15,0.5,10,2 | NRT | 91 | No NRT | 91 | 6wks | 91 | 91 | Skin irritation, nausea |
|  |  |  |  |  |  |  | / |  | Usual care | 92 | Not include | | | |
| 2003 | Wennike P | Sweden | >=15 | >=3 | gum | 2 | Gum | 65 | Placebo | 68 | 12mon | 205 | 206 | no sig.dif. |
|  |  |  |  |  |  | 4 | Gum | 140 | Placebo | 138 |  |  |  |  |
| 2003 | Glavas D | Croatia | >=1 | >=1 | patch | 7 to 21 | Patch | 56 | Placebo | 56 | 3wks | 56 | 56 | na |
| 2003 | Swanson NA | USA | 19* | 10* | patch | na | Patch | 30 | No patch | 50 | 9wks | 30 | 50 | na |
|  |  |  |  |  |  |  | Patch +bupropion | 30 | bupropion | 30 | Not included | | | |
| 2004 | Cooper TV | USA | >=10 | na | gum | 2 | Gum | 146 | Placebo | 148 | 13wks | 146 | 148 | na |
| 2005 | Batra A | Europe | >=20 | >=3 | gum | 4 | Gum | 184 | Placebo | 180 | 12mon | 184 | 180 | hiccups |
