# Supplementary material for: Effectiveness of smoking cessation therapies: a systematic review and meta-analysis
Source: BMC Public Health. 2006 Dec 11;6:300. doi: 10.1186/1471-2458-6-300 (PMC1764891; doi:10.1186/1471-2458-6-300)
Supplement: Additional File 2 — Characteristics of Bupropion RCTs. Word file displays specific study details [file 1471-2458-6-300-S2.doc]

### Additional File 2

| Author | Year | Country | Charact. of Patient  Cigarettes/day | Pack years | Bupropion Dosage  (Mg/d) | N of Participants | Control group | N of Control | Duration of TX  (Week) | Adverse events |
| --- | --- | --- | --- | --- | --- | --- | --- | --- | --- | --- |
| Zellweger J | 2005 | Europe | > 10 | 26 | 300 SR | 517 | Placebo | 170 | 7 | Insomnia  Dry mouth |
| Tonnesen P | 2003 | Europe | >10 | 30 | 300 SR | 527 | Placebo | 180 | 7 | Dry mouth |
| Tonstad S | 2003 | Europe | >10 | 49 | 300 SR | 315 | Placebo | 314 | 7 | Insomnia dry mouth nausea constipation |
| Simon JA | 2004 | USA | >20 | 39 | 300 SR | 121 | Placebo | 123 | 7 | Dry mouth gastrointestinal upset |
| Jorenby DE | 1999 | USA | >15 | 25 | 300 SR | 244 | Placebo | 160 | 9 | Insomnia  Dry mouth |
|  |  |  |  |  | 300 SR  with patch# | 245 | Placebo with Patch# | 244 |  |  |
| Hall SM* | 2002 | USA | >10 | 20 | 300 SR | 73 | Placebo | 73 | 12 | Dry mouth, constipation.  But No sig. Dif. |
| Holt S | 2005 | New  Zealand | >10 | Past 1 yr | 300 | 88 | Placebo | 46 | 7 | Insomnia |
| Hurt RD | 1997 | USA | >15 | Past 1 yr | 100 SR  150 SR  300 SR | 615 | Placebo | 153 | 7 | Insomnia  Dry mouth |
| Gonzales D | 2006 | USA | >10 | 24 | 300 SR | 329 | Placebo | 344 | 12 | cholecystitis: septic shock, headache, grand mal seizure Insomnia |
| Jorenby DE | 2006 | USA | >10 | 25 | 300 SR | 342 | Placebo | 341 | 12 | [Serious: Angioedema, Occlusion coronary artery] Insomnia,  dry mouth ,  Constipation, Sleep disorder, |
| Swanson NA | 2003 | USA | ≥5 | 10 | NA SR | 30 | No Tx. | 50 | 9 | Not mentioned |
|  |  |  |  |  | NA SR with patch# | 30 | Patch only# | 30 |  |  |
| Nides M | 2006 | USA | ≥10 | 24 | 300 SR | 126 | Placebo | 123 | 7 | Nausea, insomnia, headache, abnormal dream, taste prevention, dyspepsia, constipation, dry mouth |

*:Nortriptyine treatment groups (73 participants) were not included in our analysis; # : not included in the analysis; SR: Sustained Release.
