# Supplementary material for: Effectiveness of smoking cessation therapies: a systematic review and meta-analysis
Source: BMC Public Health. 2006 Dec 11;6:300. doi: 10.1186/1471-2458-6-300 (PMC1764891; doi:10.1186/1471-2458-6-300)
Supplement: Additional File 3 — Characteristics of Varenicline RCTs. Word file displays specific study details [file 1471-2458-6-300-S3.doc]

### Additional File 3.

| Author | Year | Country | Charact. of Patient  Cigarettes/day | Pack years | Varenicline Dosage  (Mg/d) | No. of Participants | Controlled group | No. of Intervention | Duration of TX  (Week) | Adverse events |
| --- | --- | --- | --- | --- | --- | --- | --- | --- | --- | --- |
| | Gonzales D | na | USA |  | 24 | 300 | 329 | Placebo | 344 | 12 | Insomnia | | --- | --- | --- | --- | --- | --- | --- | --- | --- | --- | --- | | na | USA |  | 25 | 300 | 340 | Placebo | 340 | 12 | Insomnia, dry mouth gastrointestinal upset | | 2006 | USA | >10 | 24 | 2 | 352 | Placebo | 344 | 12 | Abdominal pain, arterial fibrillation, pneumonia, possible stroke Insomnia |
| Jorenby DE | 2006 | USA | >10 | 25 | 2 | 344 | Placebo | 341 | 12 | Worsening vertigo, chest pain, and elevated blood pressure  Nausea, Constipation, Flatulence, Abnormal dreams, Sleep disorder |
| Nides | 2006 | USA | >10 | 24 | 0.3-2 | 377 | Placebo | 123 | 6 | Nausea, insomnia, abnormal dream, irritability, taste prevention |
| Oncken C | 2006 | USA | >10 | 25 | 1-2 | 518 | Placebo | 129 | 12 | Nausea, insomnia, abnormal dream, taste prevention, constipation, flatulence, dyspepsia, headache, somnolence |
